# Clinical and genetic factors associated with self-reported cognitive deficits in women with breast cancer: the “CAGE-Cog” study

**DOI:** 10.1186/s12885-022-10077-6

**Published:** 2022-09-19

**Authors:** Aline Hajj, Rita Khoury, Roula Hachem, Aya Awad, Souheil Hallit, Hala Sacre, Fady Nasr, Fadi El Karak, Georges Chahine, Joseph Kattan, Lydia Rabbaa Khabbaz

**Affiliations:** 1 Faculty of Pharmacy, Saint-Joseph University of Beirut, Beirut, Lebanon; 2 Laboratoire de Pharmacologie, Pharmacie Clinique Et Contrôle de Qualité Des Médicaments, Saint-Joseph University of Beirut, Beirut, Lebanon; 3 Faculty of Pharmacy, Université Laval, Québec, Canada; 4 School of Medicine and Medical Sciences, Holy Spirit University of Kaslik, P.O. Box 446, Jounieh, Lebanon; 5 Research Department, Psychiatric Hospital of the Cross, Jal Eddib, Lebanon; 6 Psychology Department, College of Humanities, Effat University, Jeddah, 21478 Saudi Arabia; 7 INSPECT-LB (Institut National de Santé Publique, d’Épidémiologie Clinique Et de Toxicologie-Liban), Beirut, Lebanon; 8 Department of Hemato-Oncology, Faculty of Medicine, Hôtel-Dieu de France Hospital, Saint-Joseph University of Beirut, Beirut, Lebanon

**Keywords:** Breast cancer, Chemotherapy, Cognitive function, FACT-Cog, *OPRM1*, Pharmacogenetics

## Abstract

**Background:**

Breast cancer patients undergoing chemotherapy treatment are at particular risk of experiencing acute cognitive impairment leading to daily challenges in decision-making and reduced quality of life and functional autonomy. The aim was to assess the relationship between clinical and genetic factors and cognitive function in a sample of patients with breast cancer undergoing chemotherapy.

**Methods:**

A cross-sectional study was carried out between November 2017 and June 2019 on women (*N* = 112) treated for breast cancer by intravenous chemotherapy at the oncology outpatient unit of Hôtel-Dieu de France Hospital, Beirut. Patients were evaluated with the 37-item Functional Assessment of Cancer Therapy-Cognitive Function (FACT-Cog). Other validated scales were also used to assess depression, anxiety, sleep disorders, pain, and fatigue. DNA was obtained by a buccal swab (FTA®technology) for genotyping of different genes (*ABCB1*, *COMT, DRD2, OPRM1, CLOCK, CRY2,* and *PER2*) using the Lightcycler®(Roche).

**Results:**

The mean age of participants was 56.04 years. Multivariable analysis, taking the four FACT-Cog subscores as the dependent variables, showed that the mean cognitive score decreased with higher depression, anxiety, and insomnia scores. Patients with university education levels had better perceived cognitive abilities than those with primary education. Moreover, carrying the G allele for the *OPRM1* polymorphism (c.118A > G;rs197791) was significantly associated with a better cognitive function compared to AA patients (B = 2.05; *p* = 0.038).

**Conclusions:**

A comprehensive oncological care plan should include a personalized assessment of all factors related to cognitive functioning in cancer patients, particularly anxiety and depression, to achieve an optimal patient outcome.

**Supplementary Information:**

The online version contains supplementary material available at 10.1186/s12885-022-10077-6.

## Background

Cancer-related cognitive impairment (CRCI) is defined as impaired memory, learning, concentration, executive function, visual-spatial abilities, and information processing [1–3]. Breast cancer patients undergoing chemotherapy treatment are at particular risk of experiencing acute cognitive impairment leading to daily challenges in decision-making, adherence to treatment, reduced quality of life and functional autonomy, and shorter survival rates [4, 5]. Hence, studies designed to evaluate cognition reported that up to 75% of women with breast cancer experience cognitive changes after a standard dose of chemotherapy [3, 6]. Several factors may account for the patients’ susceptibility to cognitive decline during a chemotherapy course, including number of therapy cycle [7], direct and indirect neurotoxicity due to treatment (progenitor cell and mitochondrial damage, immune system dysregulation) [8, 9], psychological disorders (anxiety, depression, sleep problems) [10–12], and genetic susceptibility [13–15]. Moreover, cancer-related fatigue, anxiety, depression, and insufficient sleep are associated with negative health outcomes, including impaired cognition [16–18]. As for the genetic aspect, most studies have focused on genes regulating neural repair/plasticity, such as the genes encoding the brain-derived neurotrophic factor (BDNF) [19] or apolipoprotein E (ApoE) [20]. However, other less frequently explored genetic factors might also contribute to chemotherapy-induced cognitive changes. These include the low efficiency of membrane efflux transporters at the blood–brain barrier (BBB) (such as P-glycoprotein or P-gp encoded by the *ABCB1* gene) [21], impaired neurotransmission (via dopamine pathways, known for their implication in aging and neurocognition processing, especially in the prefrontal cortex [22]), dysregulation in the brain regions known to regulate learning and memory functions (opioid system) [23–25], or dysregulation in circadian rhythms [26].

The first selected single nucleotide polymorphism (SNP) in our study included the c.3435 T > C (rs1045642) in the *ABCB1* gene encoding the P-gp. This substitution leads to a reduced P-pg expression and transport of xenobiotics to the central nervous system (CNS). Hence, patients with the T allele might have higher cognitive impairment due to a higher drug concentration in the brain, mainly because most breast cancer chemotherapy regimens have at least one cytotoxic drug that is a substrate of P-gp [27, 28]. Genes encoding the catechol-O-methyl transferase (*COMT*) and the dopamine receptor D2 (*DRD2*) were also explored. The SNP rs4680 in *COMT* is of particular interest: it causes a valine to methionine substitution (p.Val158 Met), leading to a 3- to fourfold reduced catabolic activity of COMT [29, 30]. Patients with the Met allele have a higher level of adrenaline in the prefrontal cortex (PFC) and increased dopamine binding to the D1 receptor, which enhances the stability of the dopaminergic PFC networks; these patients have a better performance in cognitive tasks (retaining information in the working memory) but a decreased cognitive flexibility while updating memory with new information [31]. As for *DRD2*, the SNP rs6277 (c.957C > T) causes a change in the mRNA folding, thus reducing the receptor stability and synthesis and the dopamine binding capacity in vivo and in vitro [32]. All these changes might lead to dysregulation in cognitive functioning in cancer patients. Despite the potential role of the opioid system in memory functions, the effects of SNPs in *OPRM1* (gene encoding the Mu-opioid receptor) on cognitive function in breast cancer patients have been rarely explored. Our team was the first to describe in palliative cancer patients that homozygous AA patients for the SNP rs1799971 (c.118A > G) of *OPRM1* had a significantly lower cognitive function than the AG patients [33]. Thus, it was decided to replicate this finding using a validated tool for cognitive function. Finally, several SNPs in genes of the circadian rhythm unexplored with cognition previously were evaluated in cancer patients: rs1801260 in *CLOCK (Circadian Locomotor Output Cycles Kaput)*, rs934945 in *PER2* (*Period circadian regulator 2*), and rs10838524 *CRY2* (*Cryptochrome circadian regulator 2*). The hypothesis was that these SNPs would induce a loss of coherence between the components of this system, which might negatively affect cognitive function [26, 34, 35].

Therefore, this study aimed to explore cognitive impairment in a group of breast cancer patients undergoing a chemotherapy course (using a validated tool for cancer patients) and evaluate the contribution of clinical, psychological, and genetic factors underlying treatment-related cognitive decline.

## Methods

### Study design and patients

The CAGE-Cog is a cross-sectional study conducted at Hôtel-Dieu de France (HDF) Hospital between November 2017 and June 2019 to evaluate the impact of Clinical And Genetic factors on cognitive function in women with primary breast cancer treated by chemotherapy.

Inclusion criteria were: adult women (> 18 years old), primary diagnosis of breast cancer (Stage 0 to IV), to be scheduled to receive a course of chemotherapy (random cycle). Exclusion criteria were: patients who refused to participate in the study, concurrent radiation/adjuvant hormone therapy, relapsed breast cancer/other types of cancer, any surgical intervention/disorder of the central nervous system (dementia, multiple sclerosis, epilepsy, Parkinson's disease, intellectual disability, and neurosurgeries) that may affect cognitive function, brain metastasis (due to their known effect on the cognitive decline) [36–38].

### Ethical approval

The ethics committee of HDF hospital approved this study (Reference: CEHDF1016, July 2017), and all patients gave their written consent prior to inclusion.

### Sociodemographic and clinical information

Several demographic and clinical data were collected (using patient interview and medical records): age, gender, weight and height (to calculate the body mass index, BMI), Body Surface Area (BSA, calculated using the Mosteller formula) [39, 40], ethnicity/nationality, marital status, education level, socioeconomic level, presence of comorbidities (diabetes, hypertension, dyslipidemia, others), current alcohol consumption (self-reported), current tobacco smoking (self-reported), and medications (other than chemotherapy). Cancer-related clinical features were retrieved from the patients' medical records, including disease stage, location of metastases (if present), chemotherapy cycle number at the time of data collection, and chemotherapy regimen.

### Cognitive function assessment and other assessments

Patients were interviewed on the day they were admitted to the oncology outpatient unit to receive chemotherapy (day 1 of chemotherapy, random cycle). The chemotherapy cycle number was recorded.

Cognitive function was assessed using the Functional Assessment of Cancer Therapy—Cognitive Function (FACT-Cog, version 3; Licensing agreement granted on November 2, 2017). This self-assessment scale consists of 37 questions divided into four subscales: 1) Perceived cognitive impairments subscale (CogPCI); 2) Perceived cognitive abilities subscale (CogPCA); 3) Comments from others subscale (CogOTH), referring to the comments of other people on cognitive function of the patient; and 4) Impact of perceived cognitive impairments on quality of life subscale (CogQOL), including disrupted normal daily functioning and working capacity. The higher the FACT-Cog score/subscore, the better the cognitive function of patients and the lower the impact on their quality of life [41].

Sleep disorders were evaluated using two screening tools, the Pittsburgh Sleep Quality Index (PSQI) and the insomnia severity index (ISI). The PSQI is a 19-item scale designed to measure sleep in seven domains during the past month: subjective quality of sleep, sleep latency, sleep duration, sleep efficiency, sleep disorders, sleep medication, and daytime dysfunction. The component scores range from 0 (no difficulty) to 3 (severe difficulty) and allow the calculation of an overall score ranging from 0 to 21 [42]. The ISI is a 7-item tool to assess the perceived severity of insomnia during the past two weeks. Questions are rated on a 5-point Likert scale from 0 (very satisfied) to 4 (not at all satisfied). Total scores range from 0 to 28, with the highest scores representing more severe insomnia [43].

Anxiety and depression were assessed using the Hospital Anxiety and Depression Scale (HADS). This self-report tool consists of two subscales designed to identify and quantify anxiety (HADS-A) and depression (HADS-D) in patients. Symptoms of the previous week are reported on a scale from 0 (not at all) to 3 (most of the time). Patients would be classified as “normal” (Score 0–7), having a “borderline anxiety/depression” (Score 8–10), or “clinical anxiety/depression” (Score 11–21) [44].

Fatigue was evaluated using three questions from the EORTC-QLQ C30 scale (European Organization for Research and Treatment of Cancer Quality of Life Questionnaire. QLQ C10: “Do you need rest?”; QLQ C12: “Did you feel weak?”; and QLQ C18: “Were you tired?”. A raw score was obtained by summing the responses to the three questions, then transformed into a score ranging from 0 to 100, according to the EORTC scoring manual. Higher scores indicated worse fatigue and a poorer QOL [45].

Finally, pain was estimated by the visual analogue scale (VAS), ranging from 0 (absence of pain) to 10 (maximum of pain) [46].

### DNA Sampling and Genotyping

DNA was obtained by a buccal swab (Whatman^®^ FTA^®^ card technology-GE Healthcare) as recommended by the manufacturer. Genotyping for selected SNPs in *ABCB1* (rs1045642), *OPRM1* (rs1799971), *COMT* (rs4680), *DRD2* (rs6277), *CLOCK* (rs1801260), *PER2* (rs934945), and *CRY2* (rs10838524) was performed using the Lightcycler^®^ 2.0 (Roche Diagnostics GmbH-Mannheim-Germany).

Supplementary Table 1 shows the polymerase chain reaction (PCR) protocol and conditions. Positive (defined by direct sequencing) and negative controls (water) were systematically included in experiments.

Physicians and investigators at the hospital assessing cognitive function and other variables were not informed of the genotyping results prior to the end of the study to minimize potential bias.

### Data and statistical analysis

The SPSS software version 25.0 was used for data analysis. Descriptive statistics were calculated for all variables in the study, including the mean and standard deviation for continuous measures and counts and percentages for categorical variables. Each of the cognitive sub-scores (CogPCI, CogPCA, CogOTH, CogQOL) was analyzed separately. The different cognitive scores did not follow a normal distribution (verified by the Kolmogorov–Smirnov test); therefore, nonparametric tests were used. The Kruskal–Wallis test was applied to compare several groups, the Mann–Whitney test to compare two groups, and the Spearman correlation test to compare two continuous variables. Multiple linear regressions were performed, taking each cognitive score as the dependent variable, respectively, and using variables that showed a *p* < 0.1 in the bivariate analysis as independent variables to reduce potential confounders. A value of *p* ≤ 0.05 was considered statistically significant.

## Results

### Sociodemographic and clinical characteristics

A total of 112 women with breast cancer were included in our study (mean age 56.04 ± 11.69 years and an average BMI of 25.9 ± 4.62 kg/m^2^). Among these patients, 8 had metastases and received palliative chemotherapy. Pain, as evaluated by the VAS, was relatively low, with a mean score of 1.68 ± 2.48 [0–9]. The average number of chemotherapy cycles was 4.45 ± 2.35 [1-12] (Table 1). More details are presented in Supplementary Table 2.

**Table 1:** Patients’ demographics and clinical characteristics (*N* = 112)^1^

		**Frequency (%)**
Marital status	Single	15 (13.4%)
	Married	94 (83.9%)
	Widowed	3 (2.7%)
Presence of metastases^2^	No	104 (92.9%)
	Yes	8 (7.1%)
Type of metastases^2^	Bone	6 (75%)
	Lung	2 (25%)
Type of chemotherapy^2^	Adjuvant	79 (71.2%)
	Neoadjuvant	24 (21.6%)
	Palliative	8 (7.2%)
	**Mean ± Standard Deviation (SD)**	**Median [25–75 Percentiles]**^**3**^
Age (years)	56.04 ± 11.69	56 [49–65]
Body Mass Index (BMI; Kg/m^2^)	25.90 ± 4.62	25.65 [23.46–28.14]
Body Surface Area (BSA; m^2^)	1.75 ± 0.16	1.74 [1.66–1.86]
Number of chemotherapy cycles	4.45 ± 2.35	4 [2–6]
Pain VAS score	1.68 ± 2.49	0 [0–3]
**Cognition**^4^		
CogPCI score	56.94 ± 14.29	58.5 [49–67]
CogPCA score	22.99 ± 5.55	24 [20–26]
CogOTH score	13.28 ± 3.17	14 [12–16]
CogQOL score	10.02 ± 4.79	10 [6–14.75]
Total FACT-Cog score	103.25 ± 23.15	107 [95–119]
**Sleep evaluation**^4^		
Insomnia Severity Index (ISI) score	10.44 ± 7.19	9 [5–15.75]
Pittsburgh Sleep Quality Index (PSQI) score	8.91 ± 4.63	9 [5–12]
**Psychological factors**^4^		
HADS-A	8.69 ± 5.25	9 [4–13]
HADS-D	7.27 ± 4.59	7 [3–11]
**Fatigue Score**^4^	42.12 ± 32.10	33.33 [11.11–66.67]

^1^Some variables did not sum up to 112 due to missing data

^2^As reported in the patients’ medical record

^3^Median and interquartile range were displayed since the variables distribution was not normal

^4^Evaluated at the oncology outpatient unit during the chemotherapy session

### Bivariate analyses

Bivariate analyses were conducted with each FACT-Cog subscale score and the total instrument score as the dependent variable in the model (Table 2).

**Table 2:** Bivariate analysis of categorical variables associated with the cognition scores

**Variable**	**CogPCI** Mean ± SD	**CogPCA** Mean ± SD	**CogOTH** Mean ± SD	**CogQOL** Mean ± SD	**Total FACT-Cog** Mean ± SD
**Education**					
Primary	57.19 ± 18.53	22.28 ± 5.95	13.12 ± 3.79	10.37 ± 5.35	102.97 ± 29.90
Secondary	57.26 ± 13.68	22.08 ± 5.19	13.13 ± 3.23	9.52 ± 4.71	102.00 ± 23.34
University	55.58 ± 13.68	24.88 ± 5.82	13.51 ± 2.85	10.78 ± 4.63	104.76 ± 21.96
* p*	0.535	0.063	0.884	0.390	0.588
**Marital status**					
Single/ widowed/ divorced	56.00 ± 14.44	24.62 ± 5.01	13.83 ± 2.23	11.66 ± 4.56	106.12 ± 22.12
Married	57.12 ± 14.33	22.68 ± 5.62	13.18 ± 3.32	9.71 ± 4.80	102.70 ± 23.42
* p*	0.632	0.315	0.712	0.09	0.803
**Diabetes**					
No	57.77 ± 13.88	23.19 ± 5.44	13.44 ± 3.07	10.18 ± 4.68	104.59 ± 22.47
Yes	49.27 ± 16.41	21.18 ± 6.54	11.82 ± 3.79	8.63 ± 5.85	90.90 ± 26.79
* p*	**0.042**	0.456	0.116	0.350	0.052
**Metastasis**					
No	56.87 ± 14.29	22.99 ± 5.64	13.34 ± 3.18	10.30 ± 4.84	103.50 ± 23.35
Yes^*^	57.88 ± 15.26	23.12 ± 4.64	12.50 ± 3.16	6.50 ± 2.07	100.00 ± 21.67
* p*	0.730	0.821	0.311	**0.022**	0.568
**Type of chemotherapy**					
Palliative regimen	57.88 ± 15.26	23.12 ± 4.64	12.50 ± 3.16	6.50 ± 2.07	100.01 ± 21.67
Adjuvant treatment	58.47 ± 12.96	23.65 ± 5.27	13.78 ± 2.70	11.00 ± 4.63	106.90 ± 20.73
Neo-adjuvant treatment	51.47 ± 17.46	20.89 ± 6.48	11.79 ± 4.11	7.75 ± 4.74	91.90 ± 28.28
* p*	0.282	0.144	**0.046**	**0.001**	**0.038**
Vinorelbine intake^**^					
No	57.00 ± 14.08	22.99 ± 5.64	13.27 ± 3.19	10.17 ± 4.80	103.44 ± 23.13
Yes	55.26 ± 21.98	23.25 ± 2.50	13.50 ± 3.11	6.00 ± 2.83	98.01 ± 26.75
* p*	0.826	0.950	0.949	0.08	0.616
Doxorubicin intake^**^					
No	56.06 ± 15.28	22.80 ± 5.85	13.01 ± 3.29	9.70 ± 4.84	101.58 ± 24.44
Yes	59.99 ± 9.77	23.68 ± 4.40	14.24 ± 2.52	11.16 ± 4.54	109.07 ± 17.14
* p*	0.430	0.839	0.072	0.212	0.188
***ABCB1* rs1045642**					
CC	56.08 ± 10.25	23.85 ± 4.18	14.09 ± 2.40	8.85 ± 4.50	102.89 ± 13.07
CT	57.18 ± 15.64	22.94 ± 5.94	13.21 ± 3.30	10.15 ± 4.70	103.49 ± 25.26
TT	56.29 ± 15.08	22.27 ± 5.65	12.92 ± 3.44	10.29 ± 5.14	101.78 ± 25.37
* p*	0.625	0.765	0.412	0.525	0.755
***COMT* rs4680**					
VV	55.00 ± 14.17	22.61 ± 5.55	13.28 ± 3.62	9.48 ± 5.10	100.39 ± 23.91
VM	57.85 ± 15.13	22.77 ± 5.92	13.56 ± 2.88	10.00 ± 4.67	104.18 ± 24.58
MM	57.22 ± 12.07	23.90 ± 4.99	12.91 ± 3.05	10.50 ± 4.86	104.54 ± 18.28
* p*	0.463	0.492	0.556	0.799	0.512
***DRD2* rs6277**					
CC	53.50 ± 14.42	23.00 ± 5.61	12.41 ± 3.69	8.70 ± 5.01	97.62 ± 25.12
CT	57.10 ± 16.08	22.62 ± 5.68	13.55 ± 3.32	9.79 ± 4.68	103.07 ± 24.75
TT	59.46 ± 10.08	23.21 ± 5.14	13.84 ± 2.33	10.71 ± 4.51	107.22 ± 16.80
* p*	0.359	0.859	0.485	0.335	0.387
***OPRM1* rs179971**					
AA	56.29 ± 14.93	22.84 ± 5.62	13.29 ± 3.31	9.30 ± 4.83	101.73 ± 24.27
AG	58.70 ± 11.50	23.39 ± 5.43	13.34 ± 2.67	12.56 ± 3.72	108.01 ± 17.81
* p*	0.743	0.519	0.671	**0.005**	0.296
***CLOCK* rs1801260**					
TT	56.04 ± 14.20	22.68 ± 6.10	13.05 ± 3.38	10.28 ± 4.77	102.06 ± 24.14
TC	56.77 ± 14.67	22.90 ± 5.93	13.36 ± 3.24	9.73 ± 4.85	102.77 ± 23.72
CC	64.00 ± 14.73	23.33 ± 3.05	16.00 ± 0.00	10.33 ± 5.50	113.67 ± 23.16
* p*	0.625	0.934	0.103	0.823	0.694
***PER2* rs934945**					
GG	55.85 ± 14.89	22.90 ± 5.97	13.58 ± 3.18	10.03 ± 5.10	102.37 ± 24.30
GA	57.42 ± 13.12	22.73 ± 4.71	12.79 ± 3.23	9.89 ± 4.23	102.84 ± 20.97
AA	66.75 ± 13.37	26.00 ± 6.48	13.50 ± 2.51	10.00 ± 5.88	116.25 ± 25.55
* p*	0.248	0.590	0.283	0.975	0.513
***CRY2* rs10838524**					
GG	55.77 ± 13.81	22.80 ± 5.97	13.32 ± 3.22	9.79 ± 4.84	101.70 ± 23.38
AG	57.77 ± 14.28	23.27 ± 5.30	13.38 ± 2.79	10.32 ± 4.67	104.74 ± 2.55
AA	56.56 ± 16.03	22.39 ± 5.48	13.05 ± 4.20	9.47 ± 5.24	101.49 ± 27.87
* p*	0.793	0.917	0.969	0.796	0.865

Numbers in bold are significant results (*p* < 0.05)

^*^Patients with metastasis were not considered as having a relapsed breast cancer (thus not excluded) because they had a primary diagnosis of metastatic breast cancer

^**^Other treatment types (chemotherapy and non-chemotherapy) did not give significant results

The results have shown that a significantly higher CogPCI score was found in patients who did not have diabetes compared to those who did. Patients taking adjuvant treatments (vs. palliative or neo-adjuvant regimens) had significantly higher CogOTH, QOL, and total scores. Patients who did not have metastases and those with the AG genotype for *OPRM1* SNP (vs. AA) had a significantly higher CogQOL score (*p* = 0.005) (Table 2).

Higher insomnia severity, pain score, anxiety, and depression levels were significantly associated with lower CogPCI, whereas higher pain score was significantly associated with lower CogPCA scores. Higher anxiety and depression scores were significantly associated with higher CogOTH. Higher insomnia severity, worse sleep quality (higher PSQI scores), higher chemotherapy cycle number, higher pain scores, and higher anxiety and depression scores were significantly associated with lower Cog QOL and total FACT-Cog scores (Table 3).

**Table 3:** Bivariate analysis of continuous variables associated with the cognition scores

Variable	CogPCI	CogPCA	CogOTH	CogQOL	Total FACT-Cog
ISI score	-0.188^c^	-0.116	-0.067	-0.350^a^	-0.250^b^
PSQI score	-0.152	-0.107	-0.119	-0.266^b^	-0.198^c^
Age	-0.109	-0.054	0.031	-0.168	-0.124
BMI	-0.098	-0.092	-0.02	-0.161	-0.120
Cycle number	-0.162	-0.02	-0.184	-0.192^c^	-0.188^c^
VAS	-0.205^c^	-0.189^c^	-0.036	-0.242^b^	-0.254^b^
Anxiety	-0.357^a^	-0.142	-0.268^b^	-0.245^b^	-0.369^a^
Depression	-0.317^b^	-0.094	-0.325^a^	-0.365^a^	-0.354^a^

^a^*p* < 0.00

^b^*p* < 0.01

^c^*p* < 0.05; *ISI* Insomnia Severity Index, *PSQI* Pittsburgh Sleep Quality Index, *BMI* Body Mass Index, *VAS* Visual Analogue Scale

### Multivariable analysis

A first linear regression, taking the CogPCI score as the dependent variable, showed that participants with higher anxiety (B = -1.06) and those with diabetes (B = -8.94) had lower CogPCI scores (Table 4, Model 1).

**Table 4:** Multivariable analysis

**Model 1: Forward linear regression taking the Cog PCI score as the dependent variable**					
**Variable**	**UB**	**SB**	***p***	**95% Confidence Interval**	
Anxiety	-1.06	-0.39	< 0.001	-1.53	-0.60
Diabetes (yes vs no*)	-8.94	-0.19	0.034	-17.17	-0.71
*Reference group; **Variables entered in Model 1:** Diabetes, ISI score, VAS score, depression, anxiety					
**Model 2: Forward linear regression taking the Cog PCA score as the dependent variable**					
**Variable**	**UB**	**SB**	***p***	**95% Confidence Interval**	
University education level compared to primary*	2.75	0.23	0.017	0.50	5.01
*Reference group; **Variables entered in Model 2:** VAS score, education					
**Model 3: Forward linear regression taking the Cog OTH score as the dependent variable**					
**Variable**	**UB**	**SB**	***p***	**95% Confidence Interval**	
Depression	-0.25	-0.36	< 0.001	-0.37	-0.13
Type of chemotherapy (adjuvant treatment vs palliative regimen*)	1.65	0.24	0.007	0.45	2.84
*Reference group; **Variables entered in Model 3:** Type of chemotherapy, depression, anxiety, doxorubicin intake, *CLOCK*					
**Model 4: Forward linear regression taking the Cog QOL score as the dependent variable**					
**Variable**	**UB**	**SB**	***p***	**95% Confidence Interval**	
Depression	-0.24	-0.23	0.012	-0.42	-0.05
Type of chemotherapy (adjuvant treatment vs palliative regimen*)	3.21	0.31	< 0.001	1.54	4.87
Insomnia severity (ISI score)	-0.17	-0.25	0.005	-0.28	-0.05
*OPRM1* (AG vs AA*)	2.05	0.17	0.038	0.11	3.98
*Reference group; **Variables entered in Model 4:** Metastasis, Type of chemotherapy, *OPRM1* SNP, PSQI score, ISI score, cycle number, VAS score, anxiety, depression, marital status, BMI					
**Model 5: Forward linear regression taking the total FACT-Cog score as the dependent variable**					
**Variable**	**UB**	**SB**	***p***	**95% Confidence Interval**	
Depression	-1.91	-0.38	< 0.001	-2.77	-1.04
Type of chemotherapy (neo-adjuvant treatment vs palliative regimen*)	-13.80	-0.25	0.005	-23.41	-4.19
*Reference group; **Variables entered in Model 5:** Type of chemotherapy, PSQI score, ISI score, cycle number, VAS score, anxiety, depression, diabetes					

***Abbreviations:**** SB* Standardized beta, *UB* Unstandardized beta

A second forward linear regression, taking the CogPCA score as the dependent variable, showed that having a university education level (B = 2.75) was significantly associated with higher CogPCA scores (Table 4, Model 2).

A third linear regression, taking the CogOTH score as the dependent variable, showed that a higher depression level (B = -0.25) was significantly associated with lower CogOTH, whereas taking an adjuvant chemotherapeutic treatment (B = 1.65) vs. a palliative regimen was significantly associated with higher CogOTH scores (Table 4, Model 3).

A fourth linear regression, taking the CogQOL score as the dependent variable, showed that a higher depression (B = -0.24) and insomnia severity (ISI; B = -0.17) were significantly associated with lower CogQOL scores, whereas taking an adjuvant chemotherapeutic treatment (B = 3.21) vs. a palliative regimen and having the *OPRM1* AG genotype vs. AA (B = 2.05) were significantly associated with higher CogQOL scores (Table 4, Model 4).

A fifth linear regression, taking the total FACT-Cog score as the dependent variable, showed that higher depression (B = -1.91) and taking a neo-adjuvant chemotherapeutic treatment (B = -13.80) vs. a palliative regimen were significantly associated with lower total FACT-Cog scores (Table 4, Model 5).

These results were adjusted over all the variables that showed a p-value of more than 0.1 in the bivariate analyses (including age and education usually controlled when considering cancer-related effects on cognition).

When forcing all the genes into each model, the results remained the same.

## Discussion

Our study explored the clinical and genetic factors associated with cognitive impairment in patients with breast cancer. The total FACT-Cog and subdomain scores were similar to those reported in other studies among breast cancer patients [20, 47], but lower than those reported in other populations after the completion of chemotherapy [10], likely due to the relatively short period of evaluation of cognitive function since the beginning of chemotherapy treatment. Regarding the genetic factors, the rs1799971 of *OPRM1* (c.118A > G) showed to be significantly associated with cognitive function; patients carrying the G variant had significantly higher CogQOL scores compared to AA patients. To the best of our knowledge, this study is the first to report such an association between the opioid system and cognitive function, using a validated tool for patient-reported (subjective) cognitive function in cancer patients. A similar finding had been previously reported in a palliative cancer group of patients but using a less specific tool for cognitive functioning (EORTC-QOL of the World Health Organization WHO) [33]. Previous studies have explored the association of dynorphin/κ-opioid receptor (κOR), but not the mu-opioid receptor, with cognitive function, showing its implication in emotion and cognition. Furthermore, it has been demonstrated that the pharmacological blockade of κOR prevented impairments of memory performance, whereas its activation was linked to cognitive decline in mice [24, 25]. Therefore, it was hypothesized that AA patients for *OPRM1*, exhibiting a greater activation of the mu-receptor, would have cognitive deficits as described with the activation of the κOR, explaining their lower cognitive performance and the subsequent impact on their quality of life. Future studies are necessary to validate these results in replication cohorts.

Besides the genetic factors, our study has shown that patients who have anxiety or depression report more cognitive impairments (lower CogPCI scores for anxiety. and lower CogOTH, CogQOL, and total FACT-Cog scores for depression). These results are in line with several previous reports showing a significant association between reported psychological distress and subjective/objective cognitive disturbances in cancer patients [10–12, 17, 48]. Indeed, psychological and emotional distress from cancer diagnosis and cancer-related treatments (chemotherapy, radiotherapy, or surgery) might cause changes in the hypothalamic-pituitary axis [49]. This hypothesis suggests that emotional states can trigger biological changes, such as the release of pro-inflammatory cytokines and biochemical alterations in neuronal plasticity, which leads to cognitive decline [8].

Another psychological factor that emerged in the multivariable analyses is the insomnia severity as evaluated by the insomnia severity index. The higher the ISI scores, the lower the CogQOL scores due to cognitive decline. This finding is similar to what was previously reported about the association between sleep disorders, cognitive dysfunction, and the detrimental impact on quality of life [50–52]. Interestingly, some researchers identified neuro-inflammatory processes [18, 53] and reduced hippocampal volumes in areas involved in working memory among patients with sleep disturbances [54].

Pain was significantly associated with cognition in the bivariate but not multivariable analyses. Previous studies exploring pain in cancer and non-cancer patients highlighted that patients reporting ongoing/persistent moderate to severe pain might be at risk for cognitive decline. A possible explanation for why this relevant factor did not remain significant in the multivariable analyses could be the relatively low pain levels reported in our study (mean VAS score of 1.68) or the relatively small sample size.

Hence, psychological disorders, pain, fatigue, sleep disorders, and cognitive dysfunction seem to be interrelated and might share a common pathophysiology, suggesting the concept of a “symptom cluster” [4, 16, 32, 43, 55].

Furthermore, patients with diabetes reported more cognitive impairment as described elsewhere [56, 57]. Several hypotheses have been suggested to explain the pathophysiology of diabetes-related cognitive dysfunction. In that context, it was found that insulin receptors are expressed in the neuronal soma and synaptic terminals, making them essential components for the preservation of memory in the hippocampus [56]. This metabolic disorder can cause a chronic inflammatory state and oxidative stress that affect neurons, inducing apoptosis and increasing cerebral damage [58]. These biochemical modifications could, therefore, potentially lead to cognitive impairments.

Having a university level of education was significantly associated with higher CogPCA scores. Several studies had previously reported the correlation between higher education and cognitive reserve [59–61]. Indeed, higher education is associated with better mental adaptation and superior coping skills following chemotherapy-induced brain damage, thus protecting against cognitive impairment [60]. A study about the impact of education level on cognitive function in breast cancer patients has reported that patients with higher education levels performed better on the verbal memory task than those who are less educated [61].

Finally, chemotherapy regimens were also associated with different subscores of the cognitive evaluation. Patients who were offered adjuvant chemotherapy following breast cancer surgery had better CogOTH/CogQOL scores than those who received a neoadjuvant or palliative regimen. A possible explanation for such a result could be the more severe/advanced cancer stages in patients who did not receive adjuvant chemotherapy.

### Limitations and strengths

Our study has several limitations related to its design. In this cohort of patients, cognitive function was not evaluated at baseline before starting any initial treatment (chemotherapy/surgery). Therefore, it was not possible to detect changes over time, and the cognitive assessment could have been biased as some patients might exhibit cognitive impairment since the beginning of their treatment. Moreover, although a validated tool for cancer patients was used, cognition was still subjectively reported and not objectively measured using clinical batteries for objective neurocognitive functioning assessment. A selection bias is also possible since patients were recruited from one hospital. An information bias is also likely since symptoms of depression, anxiety, and insomnia might have been over or underestimated by patients. Residual confounding bias might also be present since not all factors associated with cognitive function were considered in this study.

Finally, the sample size is considered relatively small for genetic analyses. However, to the best of our knowledge, this study is the first to explore several clinical and genetic factors, including genes for the circadian rhythm, not previously evaluated with cognitive function. Furthermore, multivariable analyses were performed, taking into account all confounding factors.

## Conclusion

Our findings highlight the importance of identifying clinical and genetic markers to improve the diagnosis of cognitive impairment and implement personalized medicine approaches to mitigate detrimental health outcomes in women with breast cancer. Patients with higher susceptibility to cognitive impairment could benefit from a personalized cognitive assessment as part of a comprehensive oncological supportive care plan. Management strategies, including physical activity/exercise, behavioral interventions, or other targeted pharmacological agents, that are still currently under investigation, would be interesting to implement once stronger evidence supporting their relevance is identified. Thus, further research on a larger sample is necessary to confirm our findings, allow generalization to the breast cancer population, and implement the adequate management plan.

## Supplementary Information

**Additional file 1:** **Supplementary Table 1.** The PCR protocol and conditions for *CLOCK*, *PER2*, *CRY2, COMT*, *DRD2*, *OPRM1*, and *ABCB1 *genotyping. **Supplementary Table 2.**  Patients’ demographics and clinical characteristics (*N* = 112)*.

## Data Availability

The datasets generated and/or analyzed during the current study are not publicly available due to the fact that the study is still ongoing on other cancer populations (other than breast cancer), but are available from the corresponding author on reasonable request.

## Abbreviations

ApoE: Apolipoprotein E
BBB: Blood brain barrier
BDNF: Brain-derived neurotrophic factor
BMI: Body mass index
BSA: Body Surface Area
CNS: Central nervous system
COMT: Catechol-O-methyl transferase
CogPCA: Perceived cognitive abilities subscale
CogPCI: Perceived cognitive impairments subscale
CogOTH: Comments from others subscale
CogQOL: Impact of perceived cognitive impairments on quality of life
EORTC-QLQ C30 scale: European Organization for Research and Treatment of Cancer FACIT: Functional Assessment of Chronic Illness Therapy system of Quality of Life questionnaires
FACT-Cog: Functional Assessment of Cancer Therapy-Cognitive Function
HADS-A: Hospital Anxiety and Depression Scale; anxiety subscale
HADS-D: Hospital Anxiety and Depression Scale; depression subscale
HDF: Hôtel-Dieu de France
ISI: Insomnia severity index
PCR: Polymerase chain reaction
PFC: Prefrontal cortex
P-gp: P-glycoprotein
PSQI: Pittsburgh Sleep Quality Index
SNP: Single nucleotide polymorphism
VAS: Visual analogue scale
WHO: World Health Organization
