# Supplementary material for: Clinical and genetic factors associated with self-reported cognitive deficits in women with breast cancer: the “CAGE-Cog” study
Source: BMC Cancer. 2022 Sep 19;22:996. doi: 10.1186/s12885-022-10077-6 (PMC9487123; doi:10.1186/s12885-022-10077-6)
Supplement: Supplementary file 1 — Additional file 1: Supplementary Table 1. The PCR protocol and conditions for CLOCK, PER2, CRY2, COMT, DRD2, OPRM1, and ABCB1 genotyping. Supplementary Table 2. Patients’ demographics and clinical characteristics (N = 112)*. [file 12885_2022_10077_MOESM1_ESM.docx]

Supplementary data 1

**Supplementary Table 1:** The PCR protocol and conditions for *CLOCK*, *PER2*, *CRY2, COMT*, *DRD2*, *OPRM1*, and *ABCB1* genotyping.

| ***Genes*** | **PCR** | | | | **FUSION CURVES** | |
| --- | --- | --- | --- | --- | --- | --- |
| ***CLOCK* rs1801260** | 10 s at 95˚C | 10 s at 60˚C | 15 s at 72˚C | 45 cycles | Decrease from 95 ˚C to 40˚C | Increase from 40˚C to 85˚C with a ramp of 0.2˚C/s |
| ***PER2* rs934945** | 10 s at 95˚C | 10 s at 60˚C | 15 s at 72˚C | 45 cycles | Decrease from 95 ˚C to 40˚C | Increase from 40˚C to 85˚C with a ramp of 0.2˚C/s |
| ***CRY2* rs10838524** | 10 s at 95˚C | 10 s at 60˚C | 15 s at 72˚C | 45 cycles | Decrease from 95 ˚C to 40˚C | Increase from 40˚C to 85˚C with a ramp of 0.2˚C/s |
| ***COMT* rs4680** | 10 s at 95˚C | 10 s at 60˚C | 15 s at 72˚C | 45 cycles | Decrease from 95 ˚C to 40˚C | Increase from 40˚C to 85˚C with a ramp of 0.2˚C/s |
| ***DRD2*rs6277** | 10 s at 95˚C | 10 s at 60˚C | 15 s at 72˚C | 45 cycles | Decrease from 95 ˚C to 40˚C | Increase from 40˚C to 85˚C with a ramp of 0.2˚C/s |
| ***OPRM1* rs1799971** | 10 s at 95˚C | 10 s at 60˚C | 15 s at 72˚C | 45 cycles | Decrease from 95 ˚C to 40˚C | Increase from 40˚C to 85˚C with a ramp of 0.2˚C/s |
| ***ABCB1* rs1045642** | 10 s at 95˚C | 10 s at 54˚C | 15 s at 72˚C | 45 cycles | Decrease from 95 ˚C to 40˚C | Increase from 40˚C to 85˚C with a ramp of 0.2˚C/s |

Supplementary data 2

| **Supplementary Table 2 – Patients’ demographics and clinical characteristics (N = 112)*.** | | |
| --- | --- | --- |
|  | | **Frequency (%)** |
| Nationality | Lebanese | 105 (93.8%) |
|  | Syrian | 5 (4.5%) |
|  | Other | 2 (1.8%) |
| Marital status | Single | 15 (13.4%) |
|  | Married | 94 (83.9%) |
|  | Widowed | 3 (2.7%) |
| Level of education* | Primary | 16 (14.5%) |
|  | Secondary | 61 (55.5 %) |
|  | University | 33 (30 %) |
| Profession/Work | No | 78 (69.6%) |
|  | Yes | 34 (30.4%) |
| Socioeconomic status | Low | 8 (8%) |
|  | Middle | 96 (85.7%) |
|  | High | 7 (6.3%) |
| Alcohol consumption | No | 94 (83.9%) |
|  | Yes | 18 (16.1%) |
| Smoking | No | 82 (73.2%) |
|  | Yes | 26 (23.2%) |
|  | Previous smoker | 4 (3.6%) |
| Hypertension | No | 82 (73.2%) |
|  | Yes | 30 (26.8%) |
| Diabetes | No | 101 (90.2%) |
|  | Yes | 11 (9.8%) |
| Dyslipidemia | No | 89 (79.5%) |
|  | Yes | 23 (20.5%) |
| Presence of metastases | No | 104 (92.9%) |
|  | Yes | 8 (7.1%) |
| Type of metastases | Bone | 6 (75%) |
|  | Lung | 2 (25%) |
| Type of chemotherapy | Adjuvant | 79 (71.2%) |
|  | Neoadjuvant | 24 (21.6%) |
|  | Palliative | 8 (7.2%) |
| Chemotherapy agents | Docetaxel | 80 (71.4%) |
|  | Cyclophosphamide | 59 (52.7%) |
|  | Doxorubicin | 25 (22.3%) |
|  | Carboplatin | 12 (10.7%) |
|  | 5-FU Bolus | 5 (4.5%) |
|  | Vinorelbine | 4 (3.6%) |
|  | Paclitaxel | 3 (2.7%) |
|  | Capecitabine | 1 (0.9%) |
|  | Gemcitabine | 1 (0.9%) |
| Other treatments | Folinic acid | 1 (0.9%) |
|  | Fosaprepitant | 37 (33.3%) |
|  | Setrons | 109 (97.3%) |
|  | Ranitidine | 107 (95.5%) |
|  | Atropine | 1 (0.9%) |
|  | Desloratadine | 72 (64.3%) |
|  | Promethazine | 75 (67%) |
|  | Acetaminophen | 17 (15.3%) |
|  | Methylprednisolone | 110 (98.2%) |
|  | Furosemide | 1 (0.9%) |
|  | **Mean ± Standard Deviation (SD)** | **Median [25-75 Percentiles]** ‡ |
| Age (years) | 56.04 ± 11.69 | 56 [49-65] |
| Body Mass Index (BMI; Kg/m^2^) | 25.90 ± 4.62 | 25.65 [23.46-28.14] |
| Body Surface Area (BSA; m^2^) | 1.75 ± 0.16 | 1.74 [1.66-1.86] |
| Number of chemotherapy cycles | 4.45 ± 2.35 | 4 [2-6] |
| Pain VAS score | 1.68 ± 2.49 | 0 [0-3] |
| **Sleep evaluation** | | |
| Insomnia Severity Index (ISI) score  *No clinically significant insomnia:*  *Subthreshold insomnia:*  *Clinical moderate severity insomnia:*  *Clinical severe insomnia:* | 10.44 ± 7.19  48 (42.9%)  33 (29.5%)  23 (20.5%)  8 (7.1%) | 9 [5-15.75] |
| Pittsburgh Sleep Quality Index (PSQI) score | 8.91 ± 4.63 | 9 [5-12] |
| **Cognition** | | |
| Cog PCI score | 56.94 ± 14.29 | 58.5 [49-67] |
| Cog PCA score | 22.99 ± 5.55 | 24 [20-26] |
| Cog Oth score | 13.28 ± 3.17 | 14 [12-16] |
| Cog QOL score | 10.02 ± 4.79 | 10 [6-14.75] |
| Total FACT-Cog score | 103.25 ± 23.15 | 107 [95-119] |
| **Psychological factors** |  |  |
| HADS-A | 8.69 ± 5.25 | 9 [4-13] |
| HADS-D | 7.27 ± 4.59 | 7 [3-11] |
| Fatigue Score | 42.12 ± 32.10 | 33.33 [11.11-66.67] |

* Some variables did not sum up to 112 due to missing data.

‡ Median and interquartile range were displayed since the variables distribution was not normal.
